# Mesenchymal stem cell‐derived extracellular vesicles for the treatment of acute respiratory distress syndrome

**DOI:** 10.1002/sctm.19-0205

**Published:** 2019-10-24

**Authors:** Aswin Abraham, Anna Krasnodembskaya

**Affiliations:** ^1^ Wellcome‐Wolfson Institute for Experimental Medicine Queen's University of Belfast Belfast United Kingdom

**Keywords:** cellular therapy, clinical translation, mesenchymal stem cells (MSCs), respiratory tract

## Abstract

Acute respiratory distress syndrome (ARDS) is a serious and potentially fatal acute inflammatory lung condition which currently has no specific treatments targeting its pathophysiology. However, mesenchymal stem cells have been shown to have very promising therapeutic potential, and recently, it has been established that their effect is largely due to the transfer of extracellular vesicles (EVs). EVs have been shown to transfer a variety of substances such as mRNA, miRNA, and even organelles such as mitochondria in order to ameliorate ARDS in preclinical models. In addition, the fact that they have been proven to have the same effect as their parent cells combined with their numerous advantages over whole cell administration means that they are a promising candidate for clinical application that merits further research.

**Significance statement**

Extracellular vesicles (EVs) are being actively explored as an alternative to whole‐cell therapy. Acute respiratory distress syndrome is a devastating clinical condition with high mortality rate and no pharmacological treatment; therefore, novel therapies for this condition are critically needed. This review discusses preclinical studies where the therapeutic potential of mesenchymal stem cell (MSC) EVs was investigated in models of lung injury. Evidence suggests that MSC EVs demonstrate potent protective effects mediated through a variety of mechanisms related to the transfer of EVs cargo to the recipient cells. Further research into the mechanism of action, biodistribution, standardization, and biomanufacturing is needed to facilitate clinical translation of this exciting new cell therapy product.

## 1 INTRODUCTION

### 1.1 Acute respiratory distress syndrome

Acute respiratory distress syndrome (ARDS) was first described in 1967 but its definition has changed over time^1^ with it currently being defined by the Berlin definition, which splits it into three severity levels: mild, moderate, and severe.^2^

Worldwide, ARDS affects approximately 3 million people each year and makes up 24% of all patients being ventilated mechanically in intensive care units.^3^ Currently, the 60‐day mortality rate stands at 32%^4^ and even for survivors, their long‐term quality of life and ability to do exercise is reduced. In addition, many survivors also suffer from neuropsychological deterioration.^5^

Although sepsis and pneumonia are the most common causes, ARDS can be initiated by a variety of other factors such as aspiration of gastric contents.^6^

ARDS pathophysiology is underpinned by an acute pro‐inflammatory response accompanied by damage to the alveolar epithelial‐endothelial barrier, resulting in the accumulation of protein‐rich oedema fluid in the alveoli which in turn leads to an impairment in gas exchange, leading to hypoxemia.^7^

Despite being such a serious illness with a large rate of incidence, treatment is limited to supportive measures, with no treatments directly targeting the pathophysiology of ARDS. Today, the most commonly used therapy involves mechanical ventilation as its foundation. This is combined with fluid management using diuretics to alleviate the buildup of oedema in the lungs and the patient is often placed in the prone position as this has been shown to improve oxygen perfusion in the lungs.^8^

Recently, ARDS has been recognized as a heterogeneous syndrome characterized by subphenotypes with distinct clinical, radiographic, and biologic differences, distinct outcomes, and potentially distinct responses to therapy. Biologic subphenotypes or endo‐types have been identified using plasma biomarkers, genetics, and unbiased approaches such as latent class analysis.^9^ It is this heterogeneity that is hypothesized to underlie many failures in translation of promising preclinical therapeutics to patient populations. Recently, two distinct subphenotypes have been identified within ARDS by Calfee et al, using latent class modeling in previously conducted ARDS randomized controlled trials. The subphenotypes have been termed hyperinflammatory and hypoinflammatory.^10^ The hyperinflammatory subphenotype is present in around 30% of ARDS cases and is indicated by factors such as raised levels of inflammatory biomarkers, higher prevalence of vasopressor usage and lower levels of serum bicarbonate. In addition, the hyperinflammatory subphenotype is marked by higher rates of sepsis as well as a higher mortality rate.^10^

After the existence of the subphenotypes was established, several large ARDS RC trials have been retrospectively analyzed, taking into account the presence of subphenotypes, and this has led to differences being observed in the responses to treatment between the phenotypes. For example, the preliminary study which established the subphenotypes found that low positive end‐expiratory pressure (PEEP) gave better results for mortality than high PEEP in the hypoinflammatory subphenotype whereas high PEEP gave better results in the hyperinflammatory subphenotype in the ALVEOLI trial.^10^ This was despite the original analysis of the trial showing no benefit to mortality.^11^

Further post hoc analysis of the FACCT trial^12^ has also shown significant differences in responses between phenotypes to liberal and conservative fluid management strategies.^13^ In addition, although the HARP‐2 study had previously found no significant difference in 28‐day survival in ARDS between a placebo and simvastatin,^14^ reanalysis incorporating subphenotypes found that simvastatin led to significantly higher 28‐day survival in the hyperinflammatory subphenotype.^15^

The potential of precision medicine lies in identifying novel therapeutics aimed at the subpopulation within ARDS most likely to respond and new therapies should be developed in the view of these findings.

### 1.2 Mesenchymal stem cells (MSCs) in ARDS

MSCs‐based therapy is considered as a promising approach for ARDS because of their ability to target major aspects of ARDS pathophysiology.

When used in preclinical models of ARDS, MSCs have been shown to greatly reduce inflammation and while the mechanism behind this is still not known precisely, it is known that MSCs reduce the levels of many pro‐inflammatory cytokines such as TNF‐α, IL‐1β, and IL‐6 while increasing the levels of cytokines which reduce inflammation like IL‐4, IL‐5, and IL‐10.^16^ In addition, MSCs promote bacterial clearance both directly by secreting antimicrobial peptides and proteins such as LL‐37^17^ and lipocalin^18^ as well as indirectly by activating host monocytes, macrophages and neutrophils which then phagocytose the bacteria.^19^, ^20^, ^21^, ^22^, ^23^, ^24^ Their secretion of substances such as keratinocyte growth factor (KGF) has been shown to be essential in alveolar fluid clearance and restoration of epithelial permeability.^19^, ^25^ For further information about the properties of MSCs in ARDS, consider consulting the reviews by Johnson et al^16^ and Walter et al.^26^

Due to these therapeutic effects, MSCs are being actively developed toward clinical application. MSCs have been shown to be safe in early phase clinical trials such as the START trial phase 1 and 2a.^27^, ^28^ In addition, a study known as MUST‐ARDS conducted by Athersys Inc. with a patented bone marrow derived adult multipotent progenitor cell product named “MultiStem” found a lowering of 28‐day mortality and an increase in both ventilator and ICU free days using the treatment.^29^ As well as these, the parallel trial, REALIST studying the administration of umbilical cord derived mesenchymal stem cells in ARDS is currently at the recruitment stage for phase 1 (NCT03042143).

MSCs have been proven to have an immunomodulatory effect through multiple mechanisms.^30^ Although initially, it was thought that they would promote regeneration of the injured lung tissue through engraftment and trans‐differentiation, now it has become apparent that engraftment plays little to no role in their therapeutic action.^31^

However, it is known that they modulate host cells through direct cell‐to‐cell interactions and through the release of paracrine factors including biologically active agents such as KGF,^25^ indoleamine 2,3‐dihydrogenase,^32^ and prostaglandin E2.^33^ Accumulating evidence suggests that one of the most important effectors in paracrine mechanisms of MSC effect are extracellular vesicles (EVs) which seem to be able to recapitulate the therapeutic effect of their parent MSCs.^34^

### 1.3 Issues with MSCs: Reasons to investigate MSC EVs

The need to investigate MSC EVs stems from the issues found with whole cell administration. Until recently, the tumor formation risk due to MSCs has been assumed to be fairly low owing to their short life span in vivo with many studies suggesting that they have an inhibitory effect on tumor growth,^35^ such as in liver cancer caused by hepatocyte growth factor (HGF).^36^ This makes it all the more surprising that there is now a growing field of evidence which suggests that they have the ability to promote the growth of tumors. These studies suggest that MSCs are able to travel to the site of a tumor and change the microenvironment around it, causing the stromal cells surrounding the tumor to move into the tumor itself and produce cytokines which stimulate tumor growth. Although no tumors have ever been detected which have been formed directly due to MSCs in clinical trials involving MSCs, the fact remains that they do possess ability to promote the growth of tumors and this property requires further research before MSCs can be considered safe for use in patients.^37^

Furthermore, despite their low immunogenicity, MSCs have been shown by Romieu‐Mourez et al^38^ and Chan et al^39^ to become antigen‐presenting cells, expressing MHC II antigens when stimulated by low concentrations of IFN‐λ. Although the expression on MHC II antigens occurs only within a small interval of IFN‐λ concentrations, this, alongside other factors such as low levels of TGF‐β or high cell density have been shown to cause immune responses against the MSCs which has led to them becoming rejected in some mouse models.^40^ This opens up the possibility that MSCs could trigger an immune response in patients, which may exacerbate ARDS.

Moreover, the storage of MSCs using cryopreservation requires preservatives such as DMSO. DMSO treatment produces fewer MSC colonies when plated and the survival of those colonies is reduced, especially with higher DMSO concentrations. In addition, the expression of some genes such as Bak and Bcl2 were increased when using the DMSO compared to the fresh MSCs.^41^

Furthermore, the process of freezing and thawing has been found to lower the viability of MSCs which could have an adverse effect on their therapeutic efficacy in patients.^28^

It is due to these concerns that new treatments that do not involve the administration of live cells are increasingly being investigated. It is widely accepted that MSCs provide protective paracrine effects, which are in large mediated by the secretion of EVs and therefore therapeutic potential of EV‐based therapy is being actively explored. The use of MSC‐derived EVs as a cell‐free therapeutic offer several advantages compared to MSCs: (a) EVs are non‐self‐replicating, reducing the risk of iatrogenic tumor formation; (b) EVs can be stored without DMSO at −80°C and remain biologically active; (c) MSC EVs do not express MHC I or II antigens, nor can it be induced to, allowing allogeneic transplantation; (d) EVs are less susceptible to damage by hostile environment at the site of injury (eg, hypoxia or inflammatory milieu); (e) the vesicles are small and circulate readily whereas many MSCs do not get beyond the first pass capillary bed; and (f) the dose of infused MSCs quickly diminishes post‐transplant, and it may be that the delivery of MSC‐derived vesicles can achieve a higher “dose” that circulates to a greater extent than the larger cells.

### 1.4 Definition and nomenclature of EVs

EVs are small circular structures surrounded by a phospholipid membrane which are released by cells and act as a package for various substances. When they were first discovered and for considerable time afterward, EVs were thought to be pieces of debris. It was assumed that they originate as a result of damage to the cell or due to the process of replacement of the cell membrane.^42^ However, it is now known that EVs are vital in intercellular communication as they can transport a variety of substances large distances across the body and modulate functional activities of the target cells.

As of yet, there is no consensus on the classification of EVs. However, EVs can be categorized broadly using three criteria: their size, the method by which they are formed in the parent cell, and the contents which they carry. One of the categories of EVs are “microvesicles (MV).” These are normally fairly heterogeneous, and their diameter ranges from 50 to 1000 nm.^43^ They are formed when the cell membrane projects outward from the cell and detaches, budding off the membrane, forming a closed sphere containing cytoplasm. The release of microvesicles can be stimulated by many factors such as oxidative or shear stress, hypoxia, or injury. At a molecular level, the cause of stimulation of microvesicle release depends on the cell type. In many cells, such as dendritic cells, calcium ions can act as a second messenger to stimulate the release of microvesicles, whereas in others, the phorbol ester activation of protein kinase C can have the same effect.^44^, ^45^

Another category of EVs are exosomes. In contrast to microvesicles, the diameter of exosomes is reasonably homogeneous with the diameter range being from 30 to 100 nm.^43^ Another way in which exosomes differ from microvesicles is that although microvesicles are formed by budding off from the cell membrane, exosomes have their origins within multivesicular bodies in an endosome, in which multiple exosomes are kept while inside the cell. They are then released out of the cell through exocytosis when the multivesicular bodies fuse with the cell membrane.^45^ This process is reliant on regulation of cytoskeletal changes by p53.^46^

The third category of EVs is apoptotic bodies. These are EVs which are released by cells as they are undergoing apoptosis and contain material that is about to be phagocytosed such as organelles and sections of DNA. Unlike the other EVs, these are over 1 μm in diameter.^47^

### 1.5 EV isolation, characterization, and purification

Currently, there is no globally accepted standard for the isolation, characterization or purification of EVs and methods used depend on the material from which EVs are extracted, the volume of the sample and the application of the EVs.^48^

Although EVs are often extracted from a variety of biofluids such as plasma, serum, or urine, according to a survey carried out by the ISEV (International Society for Extracellular Vesicles), the most common starting material for EV extraction used by their members was conditioned cell culture media.^49^

According to the same survey, the most common isolation method was ultracentrifugation, particularly among researchers using conditioned cell culture media. This typically involves two stages with the first stage composed of spins at increasing speeds to sediment structures which have a higher buoyant density than EVs. The second stage involves speeds of over 100 000*g* in order to sediment EVs. This is followed by washing and microfiltration of the EV suspension in order to purify the EVs.^50^, ^51^

Although this method is the one used most often, it is not without problems. While washing increases purity, the number of EVs obtained is lower.^51^ Also, factors such as centrifugation speed, type of rotor and centrifugation time have an effect on the purity, yield and sedimentation efficiency and therefore must be optimized according to the experiment, making standardization difficult.^52^, ^53^ This, combined with the fact that it cannot be scaled make it unsuitable for large scale EV isolation for therapeutic purposes.

In contrast, density gradient centrifugation, the second most widely used technique gives a greater EV purity and also higher amounts of EV proteins and RNA than ultracentrifugation.^51^ Although sucrose is the most commonly used cushion material, recently, it has been demonstrated that iodixanol can better preserve the size of the vesicles.^54^ However, this is likely not applicable in a clinical setting either due to its complexity, cost and amount of time consumed.

Ultrafiltration, the next most commonly used method separates EVs by size and is arguably a simpler process, and a viable alternative to ultracentrifugation, especially when combined with size exclusion chromatography.^55^

In addition to these techniques, precipitation of EVs through the use of various substances such as PEG (polyethylene glycol) is also used often. This has the benefit of being scalable and when combined with ultracentrifugation, gives sufficient EV purity.^56^ Hence, this is a viable method for large scale production of EVs for clinical use and has been used in one clinical study.^57^

This scalability is also seen with techniques such as size exclusion chromatography which can also be combined with ultrafiltration or ultracentrifugation to create EVs in an efficient way which has potential for standardization according to the ISEV.^34^, ^58^, ^59^, ^60^

The characterization of EVs can be done by four distinct methods according to guidelines from the ISEV.^60^ First, the cell source of the EVs (MSCs in the case of MSC EVs) should be quantified; next, the amount of EVs, derived from specific number of cells, should also be quantified. This can be achieved through techniques measuring EV number such as nanoparticle tracking analysis and flow cytometry and supplemented by quantification of the total levels of protein, lipids, or RNA.^61^ According to the MISEV2018 guidelines, the presence of EVs should be demonstrated by the analysis of at least one transmembrane protein associated to the plasma membrane (eg, CD9, CD63, CD81) and one cytosolic protein in EVs (eg, TSG101 and ALIX).

Next, the subtype to which the EVs belong should be characterized by analysis of the protein composition of the EVs using Western blotting and by analysis of nucleic acids using PCR.^61^ Single EV analysis may also be carried out using imaging techniques like transmission electron microscopy.^62^

In addition, co‐isolated components present in the sample should also be characterized as it is questioned whether these could contribute to the effect of EVs.^34^

### 1.6 Mechanisms of action of EVs

EVs interact with their target cells through receptor‐mediated binding after which they can either fuse with the cell membrane to release the contents into the cell or be taken into the cell through endocytosis, a process in which they are placed into an endocytosed vesicle.^45^

EVs can carry a variety of substances such as lipids, multiple species of RNA, various proteins including enzymes, and transcription factors and even organelles such as mitochondria. In addition to acting as complexes that essentially carry signals between cells, EVs also transfer receptors from one cell to another. This has been shown for example by Barry et al who observed that EVs were able to transfer CD41 originally made in platelets to target endothelial cells.^63^

Their ability to deliver proteins means that they can target specific mechanisms within the cell. For example, it has been shown by Sarkar et al that EVs can deliver caspase‐1 which acts to induce cell death in smooth muscle cells.^64^ Their ability to transfer mRNA and miRNA also means that they can alter the transcriptional landscapes of target cells. The delivery of mRNA to cells induces the production of proteins through mRNA translation within the target cell. In addition, they can change the epigenetic environment within cells, causing the regulation of certain genes. This has been shown using EVs isolated from murine embryonic stem cells which were applied to murine hematopoietic progenitor cells. The EVs had the effect of upregulating markers in the hematopoietic progenitor cells associated with pluripotent cells such as Oct‐4 and Nanog. In addition, the levels of mRNA for transcription factors associated with embryonic stem cells were also increased greatly and the authors believe that the pluripotency of the hematopoietic progenitor cells may have been increased. This shows that many of the characteristics of the parent cells from which EVs are extracted can be passed on to target cells through the EVs themselves, not just through the transfer of proteins but also by epigenetic means.^65^

Phinney et al^66^ reported that MSCs secrete EVs which contain functionally active mitochondria and multiple miRNAs. This finding is extremely important as ARDS often results in Multiple Organ Dysfunction Syndrome which is associated with mitochondrial dysfunction. Survivors have better mitochondrial function with preservation of ATP and biogenesis markers.^67^, ^68^ Hence, strategies aiming to protect mitochondria from injury or to increase mitochondrial biogenesis are increasingly being explored as promising therapeutic opportunities, and MSC EV‐mediated mitochondria transfer is among the most exciting.^69^

### 1.7 MSC EVs in early phase clinical trials

Multiple recent studies have presented preclinical data addressing the reparative and regenerative properties of MSC vesicles following injuries to the kidney, heart, liver, brain, lung, hind limb ischemia injury^70^ as well as various immune disorders.^71^, ^72^, ^73^ The mechanisms have been primarily mediated through the transfer of the content from the vesicles to the recipient cells, changing the function and/or phenotype. The first evidence of clinical administration of MSC EVs to patient was reported in GvHD in 2014 with promising results.^57^ Currently, there is one ongoing phase 1 study investigating the therapeutic effect of MSC EVs (exosomes and microvesicles) in type 1 diabetes (NCT02138331). However, the study has passed its completion date and the status has not been updated yet. The same team conducted a subsequent randomized, placebo‐controlled, phase 2/3 clinical study to investigate the safety and therapeutic efficacy of human cord blood‐derived EVs in inhibiting the progression of grade III and IV chronic kidney disease. The results of the trial suggest that MSC‐EV administration was safe, had a significant effect on the amelioration of overall kidney function as well as the modulation of inflammation.^74^ In addition, a phase 1 clinical trial to assess the safety and efficacy of MSCs and MSC EVs for promoting healing of large and refractory macular holes is currently at the recruitment stage (NCT03437759).

### 1.8 MSC EVs in preclinical models of ARDS

**Figure 1:**
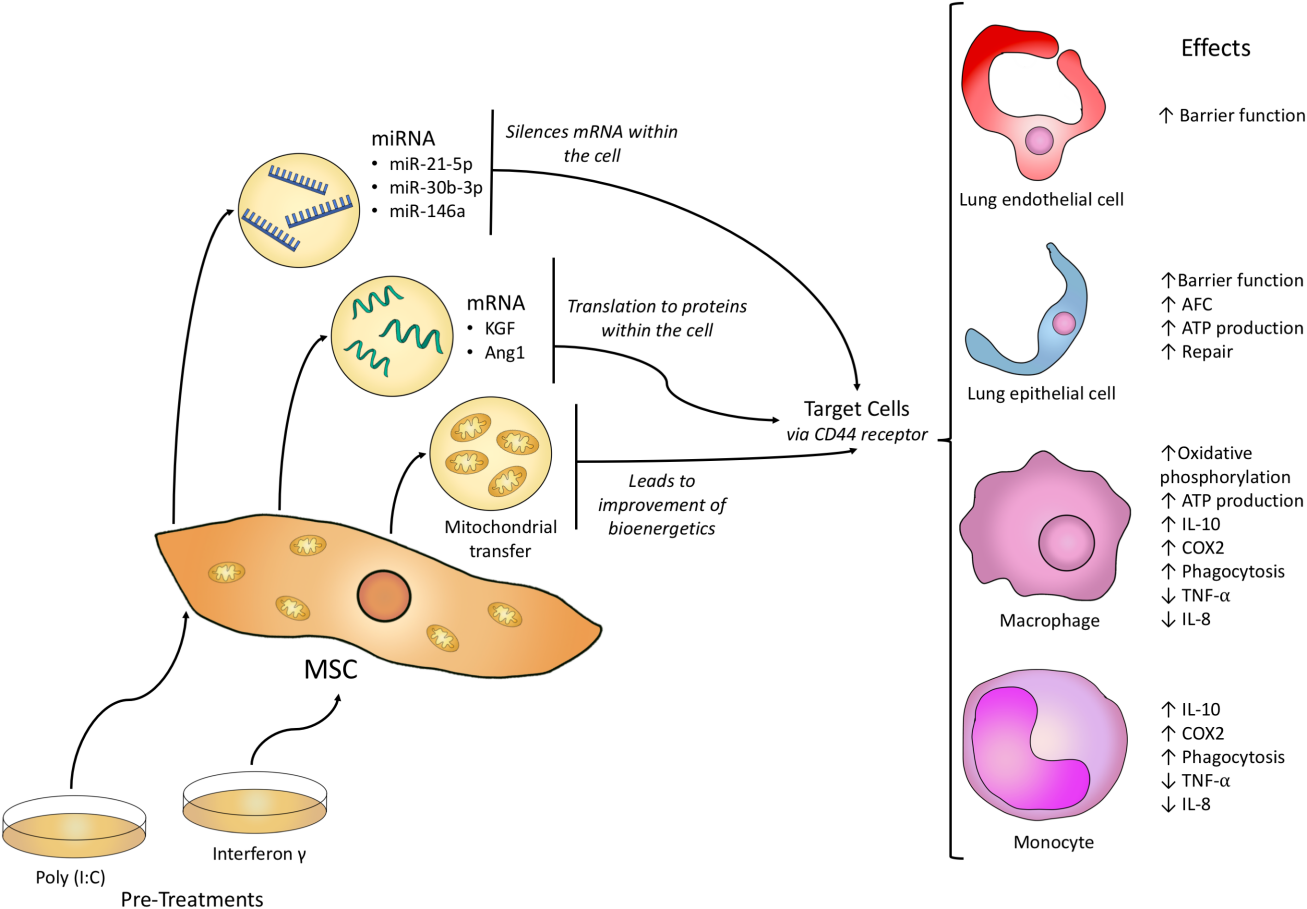
Transfer of miRNA, mRNA, and mitochondria from mesenchymal stem cells (MSCs) to different target cells by extracellular vesicles (EVs)

Although the effect of MSCs themselves in preclinical models of ARDS has been studied very well, the study of the therapeutic effect of MSC‐derived EVs in ARDS is fairly new and the knowledge base is not currently extensive (Figure 1 and Table 1).

**Table 1:** Studies investigating the effect of MSC‐derived EVs in preclinical models of ALI and ARDS

Study	Model	Treatment	Mechanism
Zhu et al (2013)^75^	Mouse ALI *E. coli* endotoxin induced ATII cells (primary culture) Injured with cytomix Ex vivo perfused human lung RAW 264.7 mouse macrophage cells	EVs from human bone marrow MSCs	↓ Inflammatory cell influx ↓ Alveolar MIP‐2, protein ↓ EVLW Restoration of protein permeability similar to MSCs Expression of KGF mRNA ↑ KGF (in both mice + ATII) ↑↑ IL‐10
Morrison et al (2017)^24^	In vitro primary human monocyte derived macrophages stimulated with LPS or BALF samples from ARDS patients Mouse model of LPS‐induced lung injury	Bone marrow MSC conditioned medium, MSC EVs, EVs were used to treat murine alveolar macrophages which were then adoptively transferred to mice	Transfer of functional mitochondria via EVs resulted in ↑Oxidative phosphorylation which led to enhanced phagocytosis and ↓TNF‐a and ↓IL‐8 secretion by macrophages in vitro and in vivo
Tang et al (2017)^77^	Mouse ALI *P. aeruginosa* induced RAW 264.7 mouse macrophage cells	EVs from human bone marrow MSCs	↓ WBC influx ↓ MIP‐2 secretion Restoration of pulmonary capillary permeability, BALF albumin level similar to MSCs Expression of Ang‐1 mRNA ↑ Alveolar Ang‐1 ↓ TNF‐α ↑ IL‐10
Gennai et al (2015)^80^	Ex vivo perfused human lung (rejected for transplant)	EVs from human bone marrow MSCs	↑ AFC rate ↓ Lung weight gain Prevention of tracheal pressure elevation ↑ Lung compliance ↓ Pulmonary artery pressure and resistance ↑ NO in perfusate ↓ pH of perfusate ↓ Elevation of lactate CD44 shown to be essential to effect
Monsel et al (2015)^78^	Mouse ALI due to severe pneumonia *E. coli* induced	EVs from human bone marrow MSCs With and without Poly (I:C) pretreatment	↓ Total bacterial load ↓ Inflammation ↓ Lung protein permeability ↑ Monocyte phagocytosis (improved even further with Poly (I:C) pretreatment) ↓ TNF‐α by LPS primed human monocytes (decreased further by Poly (I:C) EVs) Restored intracellular ATP levels in injured human ATII cells TLR3 pre‐stimulation: ↑ COX2 and IL‐10 mRNA expression in MSCs and human monocytes exposed to Poly (I:C) treated EVs Poly (I:C): ↑ IL‐10 secretion by monocytes ↓ Bacterial CFU further than by normal EVs CD44 shown to be essential to effect
Khatri et al (2018)^79^	Pig ALI Influenza virus	EVs from swine bone marrow MSCs	In lung epithelial cells: ↓Haemagglutination activity of influenza viruses ↓ Virus replication In virus‐infected pig lungs: ↓ Lung inflammation ↓ Virus replication ↓ Pro‐inflammatory cytokine production ↑ IL‐10 mRNA shown to be essential to effect
Park et al (2019)^82^	Ex vivo perfused human lung ALI *E. coli* induced Human alveolar macrophages	EVs from human bone marrow MSCs With and without Poly (I:C) pretreatment	↑ AFC rate ↓ Lug protein permeability Poly (I:C) pretreatment: ↓ Bacterial CFU ↑ Antimicrobial effect
Hu et al (2018)^83^	Human lung microvascular endothelial cells (HLMVECs) (primary culture) Injured by cytomix (IL‐1β, TNF‐α, and IFN‐γ)	EVs from human bone marrow MSCs	Protein permeability across injured HLMVECs restored Prevention of actin stress fibers formation Restoration of VE‐cadherin (adherens junction) and ZO‐1 (tight junction) Internalization of EVs found to be essential for effect Ang‐1 mRNA transfer and Ang‐1 expression shown to be essential for effect
Varkouhi et al (2019)^84^	Rat ALI *E. coli* induced In human acute monocytic leukemia cell line (THP‐1)	EVs from human umbilical cord MSCs With and without interferon γ priming	Only primed and not naïve: ↓ Alveolar‐arterial oxygen gradient ↓ Alveolar protein leak ↑ Lung mononuclear phagocytes ↓ Alveolar TNF‐α concentration ↑ Endothelial nitric oxide synthase production Both naïve and primed: ↓ Mortality ↑ *E. coli* phagocytosis ↑ Bacterial killing
Wei et al (2019)^89^	Murine Lung ischemia/reperfusion injury Murine primary pulmonary endothelial cells—hypoxia/reoxygenation model	Exosomes from murine bone marrow MSCs miR‐21‐5p agomir	↓ Lung oedema ↓ Alveolar macrophage M1 polarization ↓ HMGB1 ↓ IL‐8 ↓ IL‐1β ↓IL‐6 ↓ IL‐17 ↓ TNF‐α
Yi et al (2019)^90^	Type II alveolar epithelium cells (AEC) injured with LPS (both ex‐vivo and in vivo in a mouse ALI model)	Exosomes from bone marrow MSCs overexpressing miR‐30b‐3p	↓ SAA3 expression ↓ LPS induced AEC apoptosis
Song et al (2017)^91^	Murine Caecal ligation and puncture induced sepsis	Human umbilical cord MSCs retreated with IL‐1β	↑ Survival rate ↑ Polarization of macrophages to M2 (both compared to naïve MSCs) ↑ Exosomal miR‐146a when pretreated with IL‐1β Transfer of exosomal miR‐146a shown to be important in therapeutic effect

Abbreviations: ALI, acute lung injury; ARDS, acute respiratory distress syndrome; EVs, extracellular vesicles; MSC, mesenchymal stem cell.

One of the seminal studies on the therapeutic potential of MSC EVs for the treatment of lung injury was carried out by Zhu et al.^75^ In their experiment, they induced ARDS in mice using the intratracheal administration of endotoxin from *Escherichia coli*. EVs were isolated from bone marrow‐derived MSC‐conditioned medium using ultracentrifugation, which is the standard method followed by most studies. The effect that the EVs had on the mice was compared with the effect of MSCs. It was found that MSC EVs reduced lung inflammation and reduced oedema to the same levels as MSCs.

Interestingly, it was demonstrated that KGF mRNA was transferred from the EVs to mouse lung cells and expressed. However, the authors themselves admit that the way in which KGF concentration was determined (using ELISA) may not have been sufficient to come to this conclusion as it is not certain whether it detected only human KGF or whether it also detected mouse KGF.

Our group has found that EVs are the major component of the MSC secretome responsible for MSC modulation of macrophages in ARDS in vitro and in vivo. Mouse alveolar macrophages treated ex vivo by MSC derived EVs conferred protection in the mouse model of LPS‐induced lung injury. We have demonstrated for the first time that transfer of functional mitochondria in EVs resulted in macrophage polarization from a pro‐inflammatory toward an anti‐inflammatory phenotype through enhancement in oxidative phosphorylation.^24^

Also, we have recently^76^ demonstrated that the transfer of functional mitochondria from MSC via EVs improves mitochondrial function (membrane potential and ATP production) in primary human distal lung epithelial cells and improves their capacity to close wounds. Importantly, we also found that hypercapnia, a condition often associated with low tidal volume ventilation in ARDS, induces mitochondrial dysfunction and although the rate of mitochondrial transfer from MSCs to recipient cells is not changed, these dysfunctional mitochondria are not able to improve recipient cell bioenergetics and promote reparative capacity of the lung epithelial cells. This finding implies that MSCs may not be therapeutically beneficial in patients with ARDS who develop hypercapnia.

An experiment by Tang et al^77^ also used human bone marrow‐derived MSC EVs in vivo in mouse model. ARDS was induced using lipopolysaccharide from *Pseudomonas aeruginosa*. This experiment found that the transfer of angiopoietin‐1 (Ang‐1) mRNA is essential for the reduction of inflammation and the restoration of alveolar capillary barrier. This was shown by the fact that silencing Ang‐1 miRNA in EVs using siRNA significantly increased both the influx of neutrophils and the level of MIP‐2. Furthermore, they also studied the effect of EVs on mouse macrophages and human lung endothelial cells and found that they have immunomodulatory effects in the macrophages by suppressing TNF‐α secretion and increasing IL‐10 secretion.

Monsel et al used *E. coli* to induce pneumonia in mice.^78^ It was the first study on the effect of MSC EVs done in an infectious lung injury model. The experiment structure was very similar to that conducted by Zhu et al. In addition to testing in mice, the effect of EVs was also investigated in human monocytes and human alveolar type II cells. The results showed that EVs reduced inflammation, indicated by a 73% reduction in the influx of neutrophils and macrophages as well as a 49% reduction in the level of MIP‐2. They also showed a reduction in oedema and the permeability of endothelial‐epithelial barrier to protein. It was also shown that CD44 receptors are essential for the uptake of EVs into cells.

Khatri et al conducted an experiment investigating the effect of MSC EVs in influenza virus induced ARDS in a pig model.^79^ They found that in pigs, the replication of the viruses was reduced by the administration of EVs. They also found that there was a reduction in the death of alveolar epithelium cells. This effect was shown to be, in part, due to RNA transfer via EVs. However, this was shown by the effect being abrogated by pre‐incubation of EVs with RNase, so no specific RNA was found to mediate the effect. As in other studies, a reduction in pro‐inflammatory cytokines was also observed.

Gennai et al carried out an experiment in ex vivo perfused human lungs.^80^ These were lungs that had been rejected for transplantation and the purpose of the study was to find out if injured lungs could be improved to a potentially transplantable standard using MSC EVs. It had been known already that MSCs could restore fluid clearance in ex vivo lungs,^81^ but the effect of EVs was unknown. It was found that EV treatment reduced the level of oedema and improved the compliance of the lung.

Park et al also used EVs in an ex vivo perfused human lung.^82^ The lungs were infected by *E. coli* to induce pneumonia. In addition to using EVs extracted from untreated MSCs, they also used EVs from MSCs that were pretreated with Poly (I:C), a Toll‐like receptor 3 (TLRP3) agonist. This was done in light of previous studies which showed that pretreatment with Poly (I:C) could possibly enhance the therapeutic properties of MSCs.^78^ Although the EVs led to a significant reduction in lung protein permeability and an increase in AFC (alveolar fluid clearance), there was no significant improvement in tracheal pressure, lung compliance, or PaO_2_. In addition, a reduction in bacterial burden was only observed with Poly (I:C) pretreated MSC EVs. Although this study yielded positive results, it does not offer any investigation into the mechanisms behind the effect. In particular, the mechanism by which Poly (I:C) may be improving the effect of EVs should be explored further.

Hu et al demonstrated that EVs are able to restore barrier properties of human lung microvascular endothelial cells (HLMVECs) that were injured with cytomix (IL‐1β, TNF‐α, and IFN‐γ).^83^ It was revealed that there was an increase in the level of angiopoetin‐1 (Ang1) mRNA and protein in the injured endothelium which was treated with the EVs, and it was also found that the pretreatment of the EVs with Ang1 siRNA stopped their effect, indicating that the transfer of Ang1 mRNA from EVs plays a crucial role in the mechanism of EVs. In addition, it was also found that the internalization of EVs was required for the MSCs to produce their effect.

Although previously mentioned studies all used bone marrow derived MSCs EVs, Varkouhi et al used EVs extracted from MSCs derived from umbilical cord and found that they attenuated acute lung injury in rats.^84^ This carries significance as umbilical cord MSCs are less invasive to obtain, have a higher proliferation capacity, and can be cultured for longer.^85^

The same study compared the effect of normal EVs with those primed with interferon‐γ. Although both led to a lowering of mortality, the primed EVs were much better than normal EVs in improving multiple parameters of lung injury such as the alveolar protein leak and alveolar‐arterial oxygen gradient.

The study also identified an improvement of macrophage phagocytosis, an increase in bacterial killing, and an increased production of endothelial nitric oxide synthase as possible mechanisms. However, more detailed mechanisms of these effects remain to be elucidated. In addition, it was found that the primed EVs were larger on average than the normal EVs. However, the reason for this is unknown and requires further investigation.

The role of EV‐mediated transfer of microRNAs is also being increasingly recognized as an important mechanism of their biological effect. MicroRNAs (miRNAs or miRs) are a class of noncoding small RNAs with approximately 22 nucleotides in length. MiRNAs bind to the 3′‐untranslated region (3′‐UTR) of mRNA, resulting in either mRNA degradation or reduced protein translation. MiRNAs are implicated in the regulation of more than 60% of mammalian mRNAs, thus their shuttling in EVs represents a potent mechanism for modulation of recipient cells. In recent years, the potential involvement of miRNAs in ARDS pathophysiology has been investigated,^86^, ^87^, ^88^ providing evidence that miRNAs act as potent regulators of the inflammatory pathways. Several studies have reported that transfer of specific miRNA in MSC EVs has alleviated severity of lung injury in preclinical models.

Wei et al^89^ demonstrated that transfer of anti‐apoptotic miR‐21‐5p was responsible for the protective effect of MSC EVs in a mouse model of lung ischemia/reperfusion injury. Exosomal miR‐21‐5p reduced oxidative stress‐induced apoptosis through targeting PTEN and PDCD4 in the lung tissues.

In the recent study by Yi et al,^90^ it was found that exosomal transfer of miR‐30b‐3p resulted in inhibition of serum amyloid A3 (SAA3). miR‐30b‐3p transfer resulted in an anti‐inflammatory and pro‐reparative effect in mouse alveolar epithelial cells both in vivo and in vitro and overexpression of miR‐30‐3p in MSC exosomes resulted in an enhancement of the therapeutic effect of MSC EVs.

Song et al^91^ showed that exosomal miR‐146a contributed to the enhanced therapeutic efficacy of interleukin‐1β‐primed MSCs in a caecal ligation and puncture‐induced sepsis model. They found that miR‐146a was upregulated by IL‐1β stimulation and selectively packaged into exosomes. This exosomal miR‐146a was then transferred to macrophages, resulting in M2 polarization, and finally led to increased survival in septic mice. Therefore, modification of exosomes from MSCs with overexpression of specific miRNAs represents a promising new direction for developing therapeutic treatments for ARDS.

### 1.9 Current challenges in translation of MSC EVs to clinical practice

The main challenge with translating MSC EVs to clinical practice is that differences in EV isolation, purification, and characterization methods mean that there is often a large degree of heterogeneity in EV preparations. This is at odds with the homogeneity required for clinical application. Therefore, criteria for the standardization and optimization of EV production should be established. Proper characterization would allow further study into the differences between the efficacy of the different types of EVs. In a position paper, members of four societies (SOCRATES, ISEV, ISCT, and ISBT) propose harmonization criteria for MSC EVs to facilitate data sharing and comparison, which should help to bring the field closer toward clinical applications.^34^

This includes suggestions such as the possibility of immortalizing MSC cell lines to ensure reproducibility although more study is required into the changes that immortalization may cause. Although EV production is not stopped due to immortalization, immortalized MSCs have been found to have features such as lower plastic adherence,^92^ so the changes in EV composition needs to be examined.

Furthermore, additional studies are required to find ways to scale up the production of EVs as they are needed in large quantities to be therapeutically effective and development of GMP protocols for EV production is also essential. Moreover, although a few protocols for the biomanufacturing of exosomes have been reported,^93^, ^94^ biomanufacturing of larger microvesicles remains largely unexplored.

To use EVs as an off‐the‐shelf therapy, their stability and storage must also be examined further. In addition, the potency of the isolated EVs must be assessed using standardized disease‐specific potency assays, which are currently lacking.

Although multiple mechanisms have been discovered which modulate the function of EVs, more work also needs to be carried out to further understand the contents within EVs, especially the differences between naïve and pretreated EVs. Also, their distribution within the body after administration and the ways in which they move through endothelial barriers need further investigation.

Moreover, the establishment of subphenotypes within ARDS patients represents a major shift in the way ARDS is viewed and future therapies, including MSC EVs should be developed in line with this emerging evidence.

In addition, research so far seems to have focused mostly on bone marrow MSC EVs. Hence, other sources should also be explored.

## 2 CONCLUSIONS

In conclusion, the ability of MSC‐derived EVs to ameliorate the causal factors of ARDS in preclinical conditions, both in vivo and ex vivo, is clear. However, although studies have concluded that EVs have similar therapeutic effects to MSCs themselves, further research into the mechanisms of action of EV‐based therapeutics and manufacturing methods as well as disease‐specific potency assays is essential.

Despite these problems that must be solved, the use of EVs holds promise and merits further research due to their therapeutic potential.

## CONFLICT OF INTEREST

The authors declared no potential conflict of interest.
